# Overview of artemisinin effectiveness during outset years of its implementation in the western Brazilian Amazon

**DOI:** 10.1590/0074-02760190075

**Published:** 2019-04-29

**Authors:** Raquel de Medeiros Pinto, Vanderson de Souza Sampaio, Gisely Cardoso de Melo, Maria das Graças Costa Alecrim, Karine Mattos, Renata Trentin Perdomo, Sabrine da Costa Cordeiro, Ana Flávia Alves Parente, Lídia Raquel de Carvalho, Rinaldo Pôncio Mendes, Marcus Vinícius Guimarães Lacerda, Wuelton Marcelo Monteiro, Simone Schneider Weber

**Affiliations:** 1 Universidade do Estado do Amazonas, Programa de Pós-Graduação em Ciências Hematológicas, Manaus, AM, Brasil; 2 Fundação de Vigilância em Saúde, Manaus, AM, Brasil; 3 Fundação de Medicina Tropical Dr Heitor Vieira Dourado, Instituto de Pesquisa Clínica Carlos Borborema, Manaus, AM, Brasil; 4 Universidade do Estado do Amazonas, Programa de Pós-Graduação em Medicina Tropical, Manaus, AM, Brasil; 5 Universidade Federal de Mato Grosso do Sul, Faculdade de Medicina, Programa de Pós-Graduação em Doenças Infecciosas e Parasitárias, Campo Grande, MS, Brasil; 6 Universidade Federal de Mato Grosso do Sul, Faculdade de Ciências Farmacêuticas, Laboratório de Biociência, Alimentos e Nutrição, Campo Grande, MS, Brasil; 7 Universidade de Brasília, Brasília, DF, Brasil; 8 Universidade Estadual Paulista, Instituto de Biociência de Botucatu, Botucatu, SP, Brasil; 9 Universidade Estadual Paulista, Faculdade de Medicina de Botucatu, Botucatu, SP, Brasil; 10 Fundação Oswaldo Cruz-Fiocruz, Instituto Leônidas e Maria Deane, Manaus, AM, Brasil; 11 Universidade Federal do Amazonas, Instituto de Ciências Exatas e Tecnologia, Itacoatiara, AM, Brasil

**Keywords:** Plasmodium falciparum, artemisinin, parasite clearance, drug resistance, treatment effectiveness, cohort study

## Abstract

**BACKGROUND:**

The elimination of malaria depends on the blocking of transmission and of an effective treatment. In Brazil, artemisinin therapy was introduced in 1991, and here we present a performance overview during implementation outset years.

**METHODS:**

It is a retrospective cohort (1991 to 2002) of patients treated in a tertiary centre of Manaus, with positive microscopic diagnosis of *Plasmodium falciparum* malaria, under treatment with using injectable or rectal artemisinin derivatives, and followed over 35-days to evaluate parasite clearance, death and recurrence.

**FINDINGS:**

This cohort outcome resulted 97.6% (1554/1593) of patients who completed the 35-day follow-up, 0.6% (10/1593) of death and 1.8% (29/1593) of follow-up loss. All patients that died and those that presented parasitaemia recurrence had pure *P. falciparum* infections and received monotherapy. Considering patients who completed 35-day treatment, 98.2% (1527/1554) presented asexual parasitaemia clearance until D4 and 1.8% (27/1554) between D5-D10. It is important to highlight that had no correlation between the five treatment schemes and the sexual parasite clearance. Finally, it is noteworthy that we were able to observe also gametocytes carriage during all follow-up (D0-D35).

**MAIN CONCLUSIONS:**

Artemisinin derivatives remained effective in the treatment of *falciparum* malaria during first 12-years of use in north area of Brazil.

Malaria is a major health problem in tropical and subtropical regions of the world. According World Health Organization (WHO) 91 countries around the world present active malaria transmission, with a total of 445,000 deaths worldwide, of which 99% are caused by *Plasmodium falciparum*. In the Americas, malaria occurs in 21 countries, predominantly in South America, where four countries (Brazil, Colombia, Venezuela and Peru) account for more than 70% of cases. In Brazil, most of the malaria cases are concentrated in the Amazon states (Acre, Amapá, Amazonas, Maranhão, Mato Grosso, Pará, Rondônia, Roraima and Tocantins), an endemic area for the disease.^1^

Appropriate and early treatment is the basis of disease control, aiming at rapid cure in order to avoid the disease progress to severe forms and even death.^2^ In this fight there is a vast and efficient therapeutic arsenal, however, despite the proven efficacy treatment failures are frequent and may be the result of drug resistance or inadequate exposure to drugs, whether due to failure in prescription and dispensing, incorrect dosage and even if the patient does not adhere to the treatment.^2^ ^,^ ^3^ Moreover, resistance to antimalarial chemotherapy has been one of the major obstacle in the fight against this disease. The monitoring of the effectiveness of antimalarial drugs is a crucial component in the disease’s control, as the parasite develops mechanisms to avoid the action of antimalarial compounds.^4^

Asexual forms of *Plasmodium* are responsible for the clinical manifestation of the disease, but its transmission occurs through the ingestion of sexual forms, gametocytes, by a mosquito vector. Artemisinin-based combination therapies (ACTs) have recognised activity against immature gametocytes, but do not act against mature gametocytes.^5^ These residual gametocytes are often found in submicroscopic fractions, being a source of infection. However, microscopical examination is not sensitive enough to detect this small parasitological load and the molecular monitoring is extremely important because it detects extremely low densities of gametocytes.^6^

In Brazil, studies published in 1992 and 2010 have reported resistance of *P. falciparum* to various compounds, such as chloroquine, sulfadoxine-pyrimethamine, mefloquine, quinine and amodiaquine.^7^ ^,^ ^8^ Currently, ACT have been recommended as the first-line therapy for treating uncomplicated *falciparum* malaria in all endemic countries and failure of this *P. falciparum* therapy was not reported in Brazil.^9^ In this therapy, the possibility of selection of resistant parasites is decreased because most of the parasite load is eliminated by the rapid action of artemisinin and the residual parasitaemia is extinguished by the partner drug, which remains longer in circulation.^10^

The widespread diffusion of artemisinin treatment results in continuous pressure for the selection of the most resistant parasites and this is of great concern since there is no other pharmaceutical option as effective as artemisinin and its derivatives.^11^ ^,^ ^12^ In 2008 and 2009 studies developed on the Thai-Cambodian border, in Southeast Asia, already show a decrease in susceptibility of the *P. falciparum* to artemisinin. These studies report a delay in the parasite clearance time in the blood of some patients, which suggests a change in the parasite’s susceptibility pattern to artemisinin and is probably the first sign of artemisinin resistance.^13^ ^,^ ^14^ ^,^ ^15^ While in 2014, a report from French Guiana also point to a similar phenomenon in Latin America.^16^

However, the efficacy of ACTs against *P. falciparum* malaria and absence of genetic markers associated with artemisinin resistance have been reported,^9^ and according to Worldwide Antimalarial Resistance Network in Latin America, there is no evidence that the protozoan’s resistance has reached in Brazil. Thus, the principal objective of our study was to determine the effectiveness of *P. falciparum* treatment with five artemisinin regimens in the western Brazilian Amazon, by monitoring the parasitaemic clearance, death and recurrence during the follow-up period.

## SUBJECTS AND METHODS

*Study site* - Our study was carried out at Fundação de Medicina Tropical Dr Heitor Vieira Dourado (FMT-HVD), Manaus, in western Brazilian Amazon. FMT-HVD is a reference hospital for the diagnosis and treatment of tropical, infectious, parasitic and dermatological diseases.

*Study design* - This was a retrospective cohort study performed to assess the responses to artemisinin-based therapy regimens. Five groups of patients were constituted regarding the treatment regimens used, three of them as monotherapy (groups **1**, **2** and **3**) and two combined therapies (groups **4** and **5**); group **1**: artesunate (AS) IV 1.5 mg/kg/day until D3; group **2**: rectal artesunate suppositories (RAS), called a Rectocap™, at 50 mg/day D0 until D4 for children; group **3**: artemether (ATM) IM 1.5 mg/kg/day until D3; group **4**: artesunate IV 1 mg/kg/day followed by oral mefloquine 20 mg/kg for 12/12 hour at D2; and group **5**: artemether IM 1 mg/kg/day until D3, followed by oral mefloquine 20 mg/kg for 12/12 hour at D2. Patients with mixed malaria, concomitance of *P. falciparum* and *P. vivax*, were treated with the same therapeutic schemes described above.

The data were grouped into two 6-year periods (sexenium), to compare all the evaluated variables. Asexual and sexual parasitaemia were monitored at admission (DO) and in the standardised days of follow-up: D1 to D7, D14, D21, D28 and D35.

*Data collection* - The data were collected by reviewing all the registration forms over an 11-year period (September 1991 to December 2002) and transferred to a protocol specifically prepared for the study. The extracted data from these forms were: *Plasmodium* species, parasite numbers, age, gender, artemisinin-based therapy and leukocytes count.

*Inclusion criteria* - Cases with parasitologically confirmed malaria caused by the single-species *P. falciparum* or mixed (*P. falciparum* plus *P. vivax*), which received treatment with one of the five artemisinin-based regimens were enrolled in the study.

*Criteria of exclusion* - Patients with incomplete or illegible information, losses to follow-up and treatments with other types of antimalarial compounds or combinations were excluded of the study.

*Evaluation of the asexual and sexual parasitaemia and recurrence analysis* - The parasite densities were quantiﬁed from thick blood ﬁlms by counting the number of ring forms until 200 leukocytes were also counted, and then multiplying the number found by 30, which assumes an average number of 6,000 leukocytes/µL. During treatment, the parasitaemia was registered daily until day 7 (D0 to D7) throughout hospitalisation and, after that, weekly until day 35 (D14, D21, D28 and 35). While, the gametocytaemia was evaluated in the diagnosis (D0) and at its appearance of gametocytes among the patients who did not have gametocytes at D0.

The “day-4” parasitaemia measure was considered a surveillance tool for artemisinin tolerance, guaranteeing the current WHO-recommended cut-off value (72 hours).^3^ ^,^ ^17^

The term recurrence was used to indicate the reappearance of the asexual parasitaemia after its remission, during the 35-day follow-up. Here, we considered recurrence as recurrent asexual parasitaemia, after having observed its negativation, during the 35-day follow-up. Recurrence cases were carefully evaluated, regarding artemisinin treatment regimens, time and age groups by Kaplan-Meier analysis.

*Statistical analysis* - The enrolled patients were stratified into nine age groups (years): [a]: ≤ 9; [b]: 10 - 19; [c]: 20 - 29; [d]: 30 - 39; [e]: 40 - 49; [f]: 50 - 59; [g]: 60 - 69; [h]: 70 - 79; and [i]: ≥ 80. The distribution of patients by year of admission, gender and age range was performed by the chi-square test. Comparison of the time for clearance and recurrence regarding age range, gender and treatment was analysed by Kaplan-Meier survival curves and by the log-rank test to compare the curves. All statistical tests were 2-tailed, and significance was set up at p < 0.05. Statistical analysis was performed using SAS, version 9.2 (SAS Institute).

## RESULTS

**Fig. 1:**
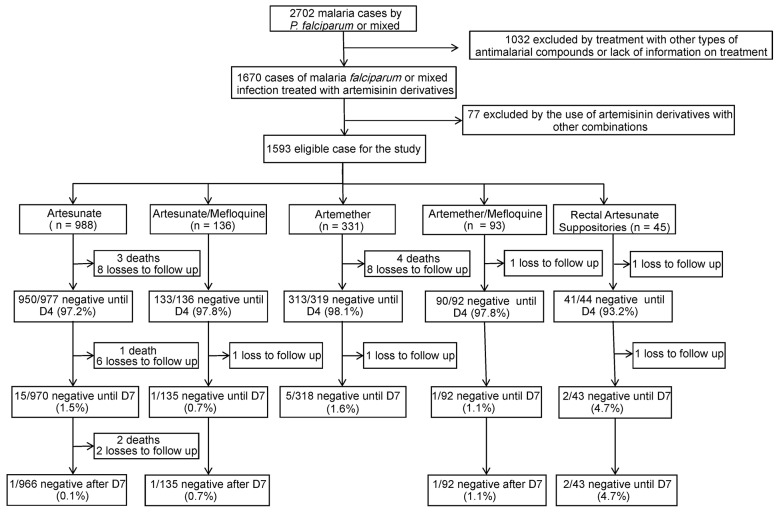
flowchart of the study.

*Patients’ characteristics and epidemiological data* - The flowchart of the study showed that the 2702 patients previously screened, 1109 of them were excluded (Fig. 1). Evaluation of all the patients showed that 97.6% (1554/1593) completed the 35-day follow-up [but 39 (2.4%) did not: 10/1593 (0.6%) of them died and 29/1593 (1.8%) lost the follow-up]. Of the 1593 eligible patients, 229 received mefloquine in combination with artemisinin, and none of them evolved to death. The artesunate or artemether in monotherapy was administered to 1364 patients, and 10 (0.7%) of them died before completing 35-day follow-up, a higher incidence than that observed in combined therapy (p = 0.009).

The number of patients with mono-infection caused by *P. falciparum* was higher than those by *P. falciparum* plus *P. vivax* (*p* < 0.0001). The distribution regarding age groups showed significant differences (p < 0.0001), paying the attention the high prevalence of patients under nine years old. These data are present in Table I. In these cases, the analysed variables were significant, except for gender (p-value = 0.32).

**TABLE I:** Comparative epidemiological profile between infection types (1991 to 2002)

Variables	*Plasmodium falciparum*	*P. falciparum* plus *P. vivax*	Total (%)	p-value*
	Number of cases			
Number of patients	1432	161	1593 (100)	< 0.0001
Age group^*a*^				< 0.0001
1	391	73	464 (29)	
2	191	18	209 (13)	
3	261	22	283 (18)	
4	156	15	171 (11)	
5	114	7	121 (8)	
6	71	7	78 (5)	
7	54	3	57 (4)	
8	13	5	18 (1)	
9	181	11	192 (12)	
Gender				0.32
male	803	83	886 (56)	
female	623	76	699 (44)	
Year				0.006
1991	28	2	30 (2)	
1992	44	4	48 (3)	
1993	103	3	106 (7)	
1994	92	5	97 (6)	
1995	114	24	138 (9)	
1996	154	18	172 (11)	
1997	119	13	132 98)	
1998	169	13	182 (11)	
1999	311	38	349 (22)	
2000	156	26	182 (11)	
2001	63	3	66 (4)	
2002	79	12	91 (6)	

*a*: the enrolled patients were stratified prospectively into nine age groups: 1 = ≤ nine years; 2 = 10-19 years; 3 = 20-29 years; 4 = 30-39 years; 5 = 40-49 years; 6 = 50-59 years; 7 = 60-69 years; 8 = 70-79 years and 9 = older than 80 years. * chi square test.

**Fig. 2:**
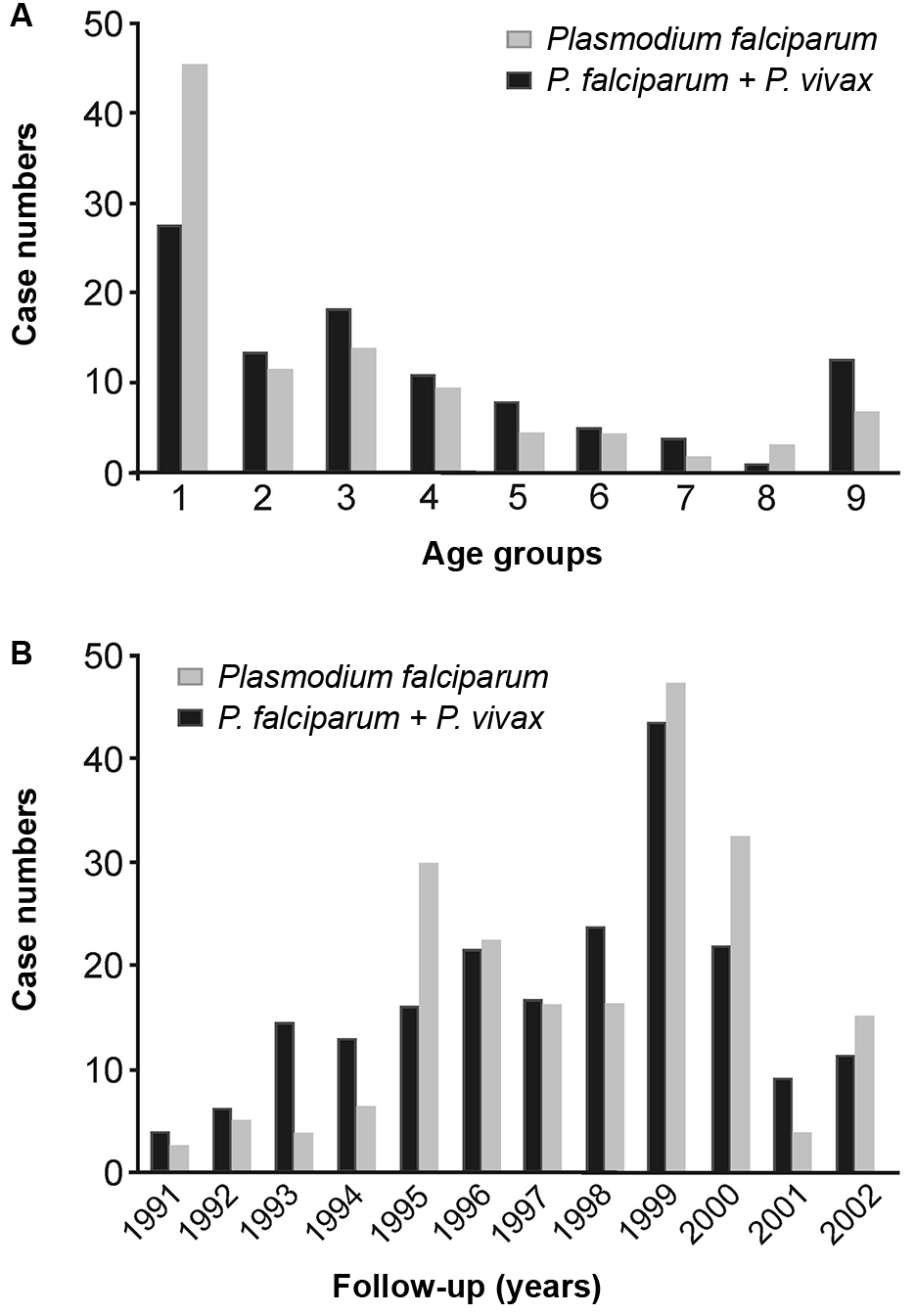
distribution of case numbers according to age range groups and year. Results were considered statistically significant when p*-*values were ≤ 0.05 by *chi-square test*. (A) (p-value < 0.0001). (B) (p-value = 0,006).

The highest malaria occurrences were observed in the youngest age groups (p < 0.0001), with a substantial increase in coinfection cases in age group **1** (one to nine years old) (Fig. 2-A); mono-infection was higher in all other age ranges. On the other hand, the coinfection showed a strong tendency to be higher (p = 0.06) in patients treated in the second sexenium than in the first one in early follow-up (Fig. 2B).

*Progress of the asexual parasite during the 35-day follow-up* - Considering patients who completed the 35-day treatment with one of five analysed artemisinin-based regimens, 98.2% (1527/1554) presented asexual parasitaemia clearance until D4 and only 1.8% (27/1554) between D5-D10 (Table II). These results revealed only three patients (0.2%) with negative parasitaemia between D7 and D10.

**TABLE II:** Asexual parasite clearance profile during 35-day follow-up (1991 to 2002)

Follow-up (days)^*a*^	AS		ASMQ		ATM		ATMMQ		RAS		Total
	No.	%	No.	%	No.	%		%	No.	%	%
D1	163	16.9	12	8.9	31	9.7	4	4.3	8	18.6	14
D2	517	53.5	69	51.1	153	48.1	35	38	25	58.1	51.4
D3	210	21.7	39	28.9	98	30.8	40	43.5	5	11.6	25.2
D4	60	6.2	13	9.6	31	9.7	11	12	3	7	7.6
Subtotal (D0-D4)	950	98.3	133	98.5	313	98.4	90	97.8	41	95.3	98.3
D5	12	1.2	1	0.7	4	1.3	1	1.1			1.2
D6	2	0.2			1	0.3			2	4.7	0.3
D7	1	0.1									0.1
(D7-D10)	1	0.1	1	0.7			1	1.1			0.2
Subtotal (D5-D10)	16	1.7	2	1.5	5	1.6	2	2.2	2	4.7	1.7
Total (D0-D35)	966	62.2	135	8.7	318	20.5	92	5.9	43	2.9	100

*a*: follow-up days: of treatment: D: day; 0-day (D0) at baseline treatment, 1-day until 7-day (D1, D2, D3, D4, D5, D6 and D7), 14-day (D14), 21-day (D21), 28-day (D28) and 35-day (D35). AS: artesunate; ASMQ: artesunate plus mefloquine; ATM: artemether; ATMMQ: artemether plus mefloquine; RAS: rectal artesunate suppositories*.*

**Fig. 3:**
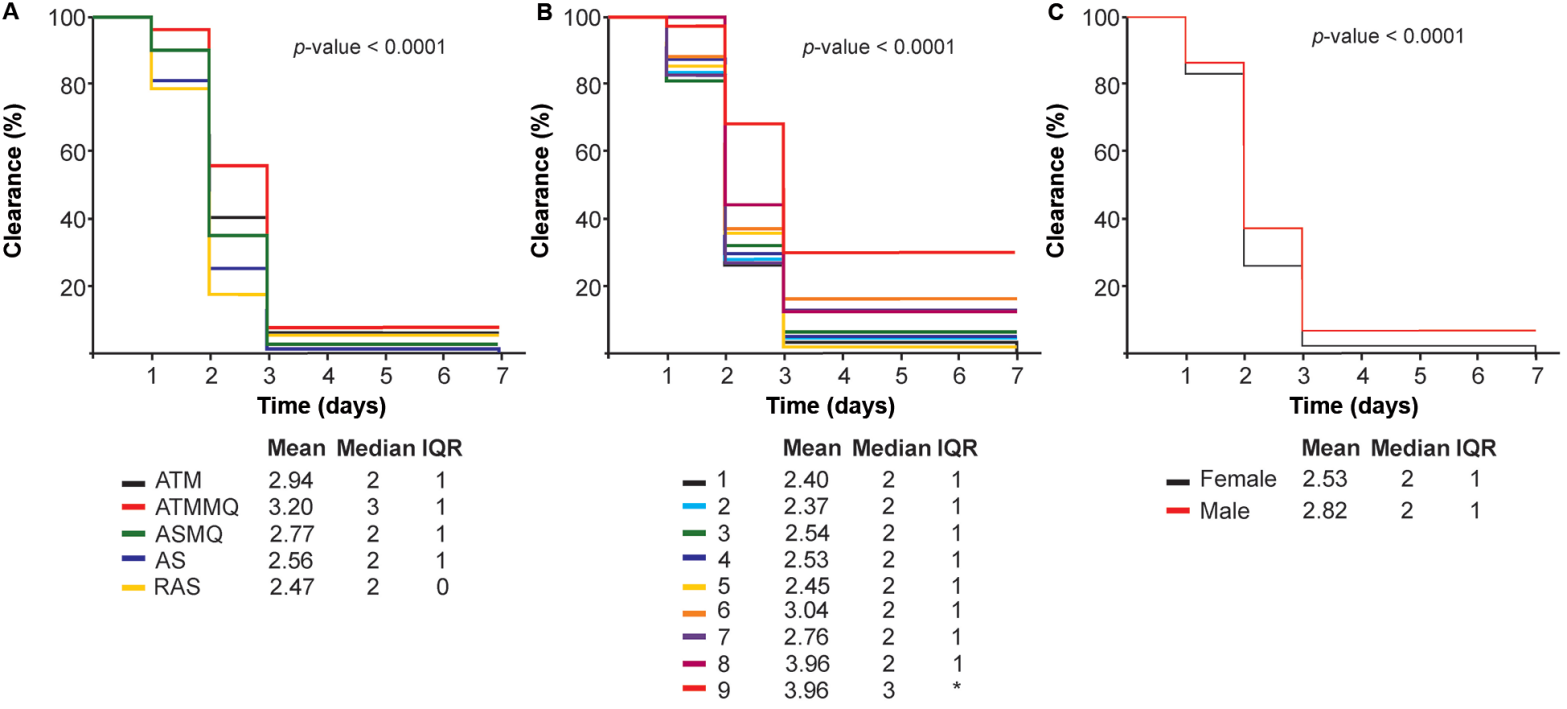
comparison of the time for clearance of asexual parasite regarding to five artemisinin-based regimens (Fig. 3A), age groups (Fig. 3B) and gender (Fig. 3C) generated by the Kaplan-Meier survival plot.

The Fig. 3 presents the comparison of the time for clearance of asexual parasite regarding to five artemisinin-based regimens (Fig. 3A), age groups (Fig. 3B) and gender (Fig. 3C) generated by the Kaplan-Meier survival plot. All analysed variables indicated statistical differences (p-value ≤ 0.05). However, when we separating cases of mono-infection and coinfection, we did not find any statistical correlation of asexual parasite clearance rates with different artemisinin-based therapies, age and gender (data not shown).

**Fig. 4:**
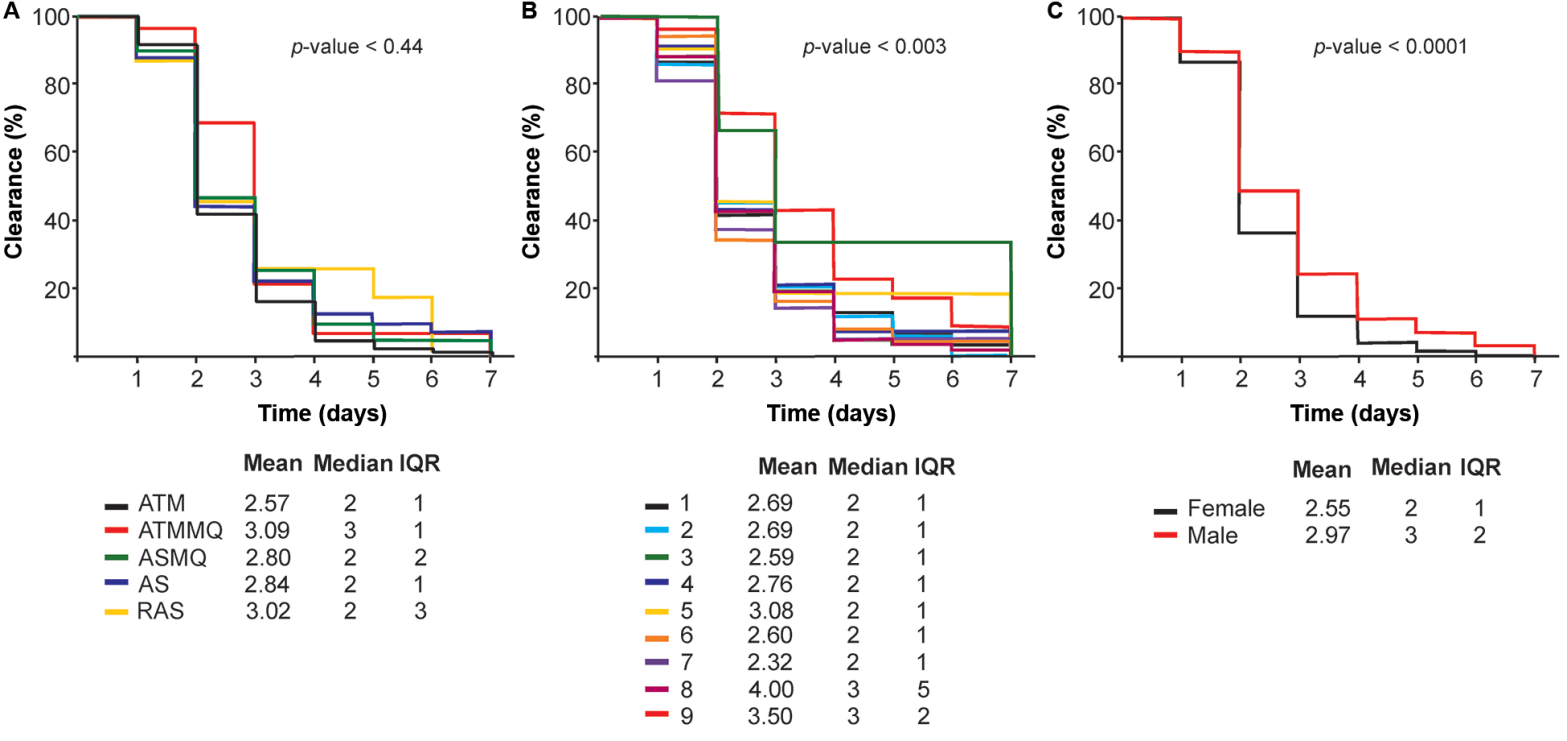
comparison of the time for clearance of sexual parasite (gametocytes) regarding to five artemisinin-based regimens (Fig. 4A), age groups (Fig. 4B) and gender (Fig. 4C) generated by the Kaplan-Meier survival plot.

*Progress of the sexual parasite during the 35-day follow-up* - The analysed sexual stage indicated prevalence of gametocytes in 47% (748/1593) of enrolled patients, 26.2 % (196/748) of whom presented gametocytes at enrollment (day 0), compared to 73.7% (552/748) who exhibited sexual stage at least once during the 35-day follow-up (Table III). Among the individuals who exhibited sexual parasitaemia, just age groups (p-value = 0.003) and gender (p-value < 0.001) revealed significant correlation with gametocyte clearance (p-values = 0.003) (Fig. 4). It is important to highlight that had no correlation between the five treatment schemes and the sexual parasite clearance during this cohort (Fig. 4A).

**TABLE III:** Prevalence of sexual parasite (gametocytes) during 35-day follow-up (1991 to 2002)

Onset of gametocytes^*a*^	Number of patients with gametocytes	
	No.	%
D0	196	12.3
D1	218	13.7
D2	195	12.2
D3	86	5.4
D4	28	1.8
D5	13	0.8
D6	5	0.3
D7	2	0.1
D14	0	0
D21	1	0.06
D28	3	0.2
D35	1	0.06
Total	748	47

a: follow-up days of treatment: D: day; 0-day (D0) at baseline treatment, 1-day until 7-day (D1, D2, D3, D4, D5, D6 and D7), 14-day (D14), 21-day (D21), 28-day (D28) and 35-day (D35).

*Parasitaemia recurrence during the follow-up* - Regarding parasitaemia recurrence in patients treated with monotherapy, 4.9% with RAS, 1.6% with AS IV and 0.3% with ATM IM demonstrated recurrence of parasitaemia during follow-up. In the other hand, patients treated with combined therapy (ASMQ and ATMMQ) did not present reappearance of the parasitaemia during the follow-up. Although the statistical analysis revealed no association with treatment regimen (p-value = 0.06), age (p-value = 0.85) or time treatment (p-value = 0.65).

## DISCUSSION

This is a 12-year retrospective analysis on the susceptibility of *P. falciparum* to artemisinin based-therapies among hospitalised patients to treatment in the Northern Brazilian region, after its introduction in 1991. Almost all hospitalised patients who completed the 35-day follow-up period presented good therapeutic response with negative parasitaemia, thus demonstrating the treatment regimen effectiveness. This finding is very important because the control and elimination of *falciparum* malaria depends not only on the blocking of transmission but also on the provision of an effective treatment.^18^ ^,^ ^19^

This study found malaria occurrence was higher in the youngest age groups, with a substantial increase of mixed malaria between one to nine years old. In this sense, is important to highlights that usually, manuscripts about treatment of hospitalised patients with malaria presents high frequency of children. However, in our study there were no predominance of children among the analysed patients, because all of them were admitted to the hospital to treatment because the recommended protocol of the Service.

Artemisinin-based therapies rapidly (~ 2 days) clear the asexual parasitaemia, which are responsible for the clinical picture, rendering this medication the choice for the treatment of *falciparum* malaria. This study showed more than 75% reduction in asexual parasitaemia clearance rates during the first three days of treatment, reaching 98.3% until D4. Delayed negative asexual parasitaemia, between D5 and D10, was observed in only 1.7% of the cases. The delay of the asexual parasitaemia clearance, by itself, can suggest resistance to this compound, but does not necessarily indicates failure of the artemisinin treatment;^13^ it is also necessary to detect associated genetic mutations. However, the K13 polymorphism had not been detected in *P. falciparum* parasites from Brazilian endemic areas.^20^ Taken together, our findings and those from Gomes et al.^20^ make us to suppose that there is no evidence of declining artemisinin-regimen effectiveness in northern Brazil during the first decade of its use. Although worldwide there have been reports of decreased artemisinin-derivative susceptibility,^21^ ^,^ ^22^ sensitivity of *P. falciparum* and treatment effectiveness remain high in Brazil.^23^ It is important to register that the control of the clearance of the asexual parasitaemia is a tool to evaluate the emergence of *P. falciparum* resistance to antimalarial compounds.

Although artesunate monotherapy was highly effective in clearing asexual blood-stage parasitaemia, a good response was also observed with the ASMQ treatment. Similar clearance to this combination therapy was previously reported in the Peruvian Amazon.^24^ In addition, Ladeia-Andrade et al.^9^ defined the use of ASMQ fixed-dose combination as the first-line regimen to treat *P. falciparum* in the Juruá Valley, a Brazilian region with high transmission of *P. falciparum*, where the authors showed no molecular evidence of resistance to AS and MQ during the 2010 to 2013 period.

It is worth mentioning that these results reflect the first decade of artemisinin-based therapy use in northern Brazil, and current monitoring should be performed to evaluate the present situation in the region. Studies using artemether^25^ and artesunate^26^ monotherapy in Suriname, published in 2013 and 2016 respectively, a neighboring region from Brazil, raised the suspicion of resistance to this compound.

In addition, several studies have reported *P. falciparum* resistance to artemisinin derivatives in southeast Asia, which is of great concern, as there is no alternative as effective as artemisinin, and the spread of resistance throughout the world would be catastrophic.^17^ ^,^ ^25^ This study compared the efficacy of artemether-lumenfantrine, evaluating the clearance of parasitaemia up to D3, with another study conducted in the period 2005-2006 to detect the appearance of resistance to artemether. Findings may represent emerging resistance of *Plasmodium* to artemether in a country outside the emerging region of resistance, Asia, which causes great concern because it is an area with a history of malaria infection, and also because it is a gold mining area.^25^

Although the main focus is the asexual parasitaemia, because it is responsible for the clinical symptoms of the disease, we also analysed the presence at admission and the emergence of sexual forms during the follow-up. The gametocyte is the transmission form from human host to mosquitoes.^6^ Therefore, the understanding of the influence of gametocytaemia and gametocidal properties of antimalarial compounds is of great relevance for actions aimed at reducing malaria transmission. Herein, we observed a relatively high proportion, 47% (748/1,593), of individuals with gametocytes, but there was no correlation between the different treatment regimens and sexual parasitaemia clearance. On the other hand, overall, 12.3% of enrolled patients had gametocytaemia at admission, and this gametocyte presence at early phase of treatment (day 0) seems to influence the malaria clearance (data not show).

It is noteworthy that in our cohort we were able also to observe sexual parasitaemia after D3 until D35. Artemisinin and its derivatives do attenuate the growth of young parasites *in vitro* and increase the clearance of ring forms *in vivo*. The artemisinin derivatives are a first-line antimalarial compounds that act only on the early stage of the gametocytes. Thus, in combined therapies the sexual parasitaemia clearance depends on mefloquine, since this antimalarial compound is cleared slowly as its half-life elimination is two to three weeks.^27^ This finding can explain the increased gametocyte carriage during treatment, to be removed in the third and subsequent post-treatment replication cycles.^28^ So, as gametocytes are the forms that are taken up by and infect the anopheline mosquitoes, to maintain the *Plasmodium* life cycle, artemisinin and its derivatives do not prevent this transmission.^29^ This is an important finding for public health, as the effect of antimalarial compounds in reducing gametocyte carriage plays a role in malaria control. For information purposes, although primaquine was not evaluated in this study because it was an exclusion criteria, during the period of the study of this cohort, there was already a national recommendation for its use with aim of eliminating the gametocytes.

Several factors may have contributed to recurrence, observed only in patients receiving artemisinin-derivative monotherapy. Noncompliance could explain these treatment failures, however the dispensing of drugs was assisted because the enrolled patients were hospitalised during their follow-up. In addition, the deaths were observed only in the group of patients with pure *P. falciparum* infection receiving artemisinin-derivative monotherapy. Thus, our results suggest that the combined therapy with mefloquine prevented recurrences and deaths, possibly because mefloquine remains longer in the bloodstream due to its greater elimination half-life, in contrast to artemisinin derivatives that are rapidly eliminated (half-life about 16 hours).

Among the patients who did not progress to death, 93% presented negative asexual parasitaemia after treatments. Differently, all patients who progress to death presented positive asexual parasitaemia until the last day of life and died until D7. So, even though the parasitaemia at D0 was lower in patients who died (4,080/µL) than those who surveilled (15,647/µL), it had been persistent. In addition, 30% of patients who died (3/10) presented gametocytes at admission compared to all enrolled patients (12.3%).

Co-infection with *P. vivax* was detected in 10% of the enrolled patients, which increased over the years, since a greater number of cases were observed in the second sexenium. In the last decade, the world has seen a significant decline in the number of malaria cases, accompanied by an increase of malaria *vivax* / malaria *falciparum* ratio. Mohapatra et al.^30)^ demonstrated that *P. vivax* infection may be protective when coexisting with *P. falciparum*, with less severe disease and a lower level of parasitaemia than it was observed in infections caused only by *P. falciparum*.^30^

*Ethics approval and consent to participate* - The study was in accordance with the ethical standards of the declaration of Helsinki and approved by the institution’s ethics committee (CEP/FMT-HVD CAAE number: 46481215.2.0000.0005).

*In conclusion* - In spite of the limitations, because it is a retrospective study involving observation for a long period of time and with information retrieved from medical record books, this study is the largest series of cases with patients treated with artemisinin-based therapies, which have already been described in the Amazon Region. This study showed stable levels of *P. falciparum* sensitivity to artemisinin derivatives in the Amazon Region over a period of 12 years. In conclusion, our findings do not evidence the occurrence of *P. falciparum* resistance to artemisinin derivatives in the assessed period; however, routine monitoring of artemisinin should continue, so that the first signs of resistance are detected early.
